# Towards a new generation of agricultural system data, models and knowledge products: Design and improvement

**DOI:** 10.1016/j.agsy.2016.10.002

**Published:** 2017-07

**Authors:** John M Antle, Bruno Basso, Richard T Conant, H Charles J Godfray, James W Jones, Mario Herrero, Richard E Howitt, Brian A Keating, Rafael Munoz-Carpena, Cynthia Rosenzweig, Pablo Tittonell, Tim R Wheeler

**Affiliations:** a Oregon State University, United States; b Michigan State University, United States; c Colorado State University, United States; d University of Oxford, United States; e University of Florida, United States; f CSIRO, Australia; g University of California-Davis, United States; h NASA/Columbia University, United States; i National Institute of Agricultural Technology, Argentina; j University of Reading, United Kingdom

**Keywords:** Agriculture, Systems, Models, Data, Knowledge products, Next generation

## Abstract

This paper presents ideas for a new generation of agricultural system models that could meet the needs of a growing community of end-users exemplified by a set of Use Cases. We envision new data, models and knowledge products that could accelerate the innovation process that is needed to achieve the goal of achieving sustainable local, regional and global food security. We identify desirable features for models, and describe some of the potential advances that we envisage for model components and their integration. We propose an implementation strategy that would link a “pre-competitive” space for model development to a “competitive space” for knowledge product development and through private-public partnerships for new data infrastructure. Specific model improvements would be based on further testing and evaluation of existing models, the development and testing of modular model components and integration, and linkages of model integration platforms to new data management and visualization tools.

## Highlights

• NextGen data and models could accelerate innovation needed to achieve sustainable systems and meet global food security objectives.
• We envision new data and models compatible with new knowledge products needed to support user-defined information needs.
• Our design strategy involves “pre-competitive” and “competitive” spaces for data, model and knowledge product developments.
• We identify potential advances for disciplinary model components and their integration to address key Use Cases.

## 1 Introduction

The idea of creating a new generation of agricultural system data, models and knowledge products (NextGen) is motived by the convergence of several powerful forces. First, there is an emerging consensus that a sustainable and more productive agriculture is needed that can meet the local, regional and global food security challenges of the 21st century. This consensus implies there would be value in new and improved tools that can be used to assess the sustainability of current and prospective systems, design more sustainable systems, and manage systems sustainably. These distinct but inter-related challenges in turn create a demand for advances in analytical capabilities and data. Second, there is a large and growing foundation of knowledge about the processes driving agricultural systems on which to build a new generation of models (Jones et al., 2017-in this issue B). Third, rapid advances in data acquisition and management, modeling, computation power, and information technology provide the opportunity to harness this knowledge in new and powerful ways to achieve more productive and sustainable agricultural systems (Janssen et al., this issue).

Our vision for the new generation of agricultural systems models is to accelerate progress towards the goal of meeting global food security challenges sustainably. But to be a useful part of this process of agricultural innovation, our assessment is that the community of agricultural system modelers cannot continue with business as usual. In this paper and the companion paper on information technology and data systems by Janssen et al. (this issue), we employ the Use Cases presented in Antle et al. (this issue A), and our collective experiences with agricultural systems, data, and modeling, to describe the features that we think the new generation of models, data and knowledge products need to fulfill this vision. A key innovation of the new generation of models that we foresee is their linkage to a suite of knowledge products – which could take the form of new, user-friendly analytical tools and mobile technology “apps” – that would enable the use of the models and their outputs by a much more diverse set of stakeholders than is now possible. Because this new generation of agricultural models would represent a major departure from the current generation of models, we call these new models and knowledge products “second generation” or NextGen.

We organize this paper as follows. First, we discuss new approaches that could be used to advance model development that go beyond the ways that first generation models were developed, and in particular, the idea of creating a more collaborative “pre-competitive space” for model development and improvement, as well as a “competitive space” for knowledge product development. Then we describe some of the potential advances that we envisage for the components of NextGen models and their integration. We also discuss possible advances in model evaluation and strategies for model improvement, an important part of the approach. Finally, we discuss how these ideas can be moved from concept to implementation.

## 2 Designing next generation models

### 2.1 A demand-driven, forward-looking approach

A first step towards realizing the potential for agricultural systems models is to recognize that most work has been carried out by scientists in research or academic institutions, and thus motivated by research and academic considerations more than user needs. A major challenge for the development of a new generation of models that is designed to address user needs, therefore, is to turn the model development process “on its head” by starting with user needs and working back to the models and data needed to quantify relevant model outputs.

The NextGen Use Cases presented in Antle et al. (this issue A) show that most users need whole-farm models, and particularly for small-holder farms in the developing world, models are needed that take into account interactions among multiple crops and often livestock. Yet, many agricultural systems models represent only single crops and have limited capability to simulate inter-cropping or crop-livestock interactions. Why? One explanation is that many models were developed in the more industrialized parts of the world where major commodity crops are produced. Another explanation is that models of single crops are easier to create, require less computational resources, and are driven by a smaller set of data than models of crop rotations, inter-crops or crop-livestock systems. Additionally, researchers are responding to the incentives of scientific institutions that reward advances in science, and funding sources that are more likely to support disciplinary science. Component processes within single crops, or single economic outcomes, are more easily studied in a laboratory or institutional setting, and may result in more publishable findings. Producing useful decision tools for farmers or policy decision-makers is at best a secondary consideration in many academic settings.

The need for more integrated, farming-system models has been recognized by many researchers for several decades, for example, to carry out analysis of the tradeoffs encountered in attempts to improve the sustainability of agricultural systems (Kanter et al., 2016). For example, Antle and Capalbo (2001) and Stoorvogel et al. (2004) proposed methods for linking econometrically estimated economic simulation models with biophysical crop simulation and environmental process models. Giller et al. (2011) describe a complex bio-physical farming system modeling approach, and van Wijk et al. (2014) review the large number of studies that have coupled bio-physical and economic models of various types for farm-level or landscape-scale analysis. More recent work by AgMIP has developed software tools to enable landscape-scale implementation of crop and livestock simulation models so that they can be linked to farm survey data and economic models (Porter et al., 2015). While these examples show that progress has been made in more comprehensive, integrative approaches to agricultural system modeling, these modeling approaches are more complex and have high data demands, thus raising further challenges to both model developers and potential users. As we discuss below, methods such as modularization may make it possible to increase model complexity while having models that are relatively easy to understand and use. Other methods, such as matching the degree of model complexity to temporal and spatial scales, also can be used. Section 3.8 further discusses issues of model complexity and scale.

While it is clear that model development needs to be better linked to user needs, it is also important to recognize that science informs stakeholders about what may be important and possible. Who imagined even a few years ago that agricultural decision support tools would use data collected by unmanned aerial vehicles linked to agricultural systems simulation models? So while model and data development need to be driven by user-defined needs, they must also be forward-looking, using the best science and the imaginations of creative scientists.

### 2.2 An open pre-competitive space for model development linked to a competitive space for knowledge product development

**Fig. 1:**
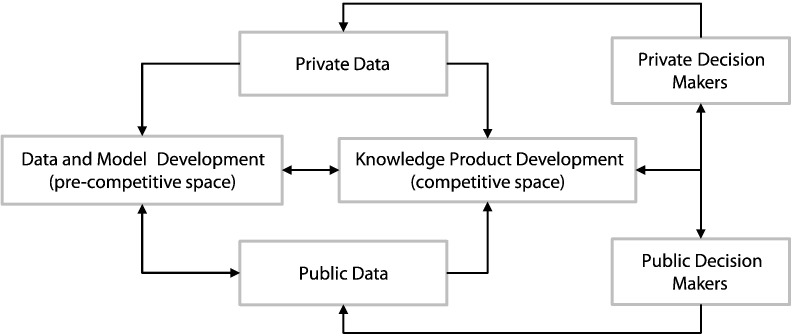
Linkages between the pre-competitive space of model and data development and the competitive space of knowledge product development.

As Jones et al. (2017-this issue A) describe in their paper on the historical development of agricultural systems models, existing models evolved from academic agronomic research. While there was a sense that “decision support” was important, the model developments nevertheless began with research tools that were motivated primarily to better understand basic processes and effects on system performance. As long as model development is motivated primarily by academic and research outcomes, it will remain only loosely connected to user needs. Therefore, to re-orient model development towards user needs, a new set of institutional arrangements and incentives is needed. Fig. 1 presents a diagram of how these new arrangements might be organized. The figure shows the linkages between a “pre-competitive space” of basic science and model development, and the “competitive space” of knowledge product development. The concept of “pre-competitive space” grew out of the efforts of the pharmaceutical industry to collaborate on basic research while competing in product development. The arrows between these two “spaces” point both ways to represent the inevitable and important give-and-take.

**Fig. 2:**
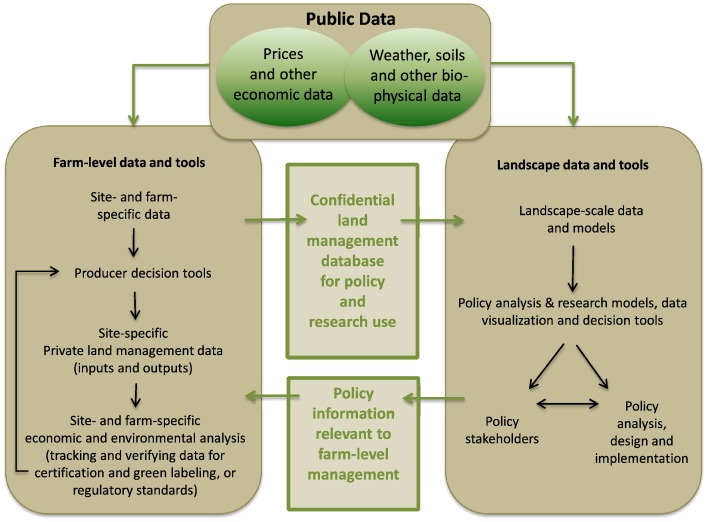
Linkages between data and decision tools at farm and landscape scales.

The model development approach that now exists is largely missing the competitive space component shown in Fig. 1. To the extent that such a competitive space does exist, it is in the private sector where proprietary management support is being provided, and linkages in Fig. 1 from competitive knowledge product development back to data and model development are largely missing. In Fig. 2 we show how this link from private decision makers to models and public data could be made by connecting on-farm decision support tools to databases that could be used for model development and analysis (see the paper by Capalbo et al., 2017-in this issue, for elaboration of these ideas).

(source: Antle et al., 2014a)

Facilitating a pre-competitive environment is likely to require innovations in the way research organizations operate, and may need to involve public-private partnerships (PPPs) that clearly delineate boundaries and roles in creating specific NextGen products. PPPs are one way that science and industry can collaborate to generate new applied knowledge that can feed into the creation of new business and services. In PPPs it is common that both private and public partners provide funding and jointly formulate the research questions that can subsequently be tackled by research institutes and universities. There are a number of challenges in structuring PPPs. For example, in the European Union PPPs have been regulated to avoid unfair competition. The EU regulations stipulate that there always has to be more than one private partner involved and intellectual property rights of the knowledge developed (e.g., tools, models, articles, methods) belong to the research partner, which can then license the use to private partners for commercial purposes.

An important aspect for a NextGen community of practice is openness. Open here means: first, inviting and engaging others to join and become involved; second, being ready to set priorities jointly with a broader stakeholder community (i.e. research programming, private partners, policy partners, non-governmental organizations); and third, being transparent for scientific and public scrutiny of methods, tools and results through not-solely scientific venues. Only a few of the agricultural systems models and economic models now in use can be said to be “open” in the sense that both the model equations and programming code are fully documented and freely available to the community of science. Establishing an open approach consistent with the principles of good science, including sufficient documentation and sharing of code to allow replication of results with reasonable effort, should be a priority of the practitioner community. Such an approach would facilitate model improvement through peer review, model inter-comparison and more extensive testing, new modes of model improvement and development such as crowd sourcing, and education of the next generation of model developers and users.

Creating this open approach will also raise challenges related to incentives and intellectual property that would need to be addressed. The recent experience with the Agricultural Model Inter-comparison and Improvement Project (AgMIP; Rosenzweig et al., 2013), a new community of science dedicated to an open approach, suggests that researchers are now more willing to participate, but also has identified some of the challenges to an open collaborative approach. For example, obtaining funding for collaborative activities creates coordination issues among research institutions and funding agencies that need to be addressed.

Another advantage of an open approach is that it will encourage the emergence of competing models and modeling approaches, rather than a single “super-model.” One dominant “super-model” could eventually emerge, but the only way to know that such a model is desirable is to allow a multi-model environment to flourish. We also expect to see alternative approaches emerge as modelers tackle challenging features such as representation of heterogeneity and dynamics and linkages across scales. For models to be tractable, tradeoffs have to be made, and an open approach is needed to facilitate the testing of alternative solutions.

There are important examples of recent efforts at creating a more open approach to agricultural model development. The bio-economic farm model FSSIM (Janssen et al., 2010) was made available as open source in 2010 after completion of its main project-related development and published with a license that allowed further use and extension. It is notable that the open sourcing of the model was combined with training sessions, but this did not lead to spontaneous community uptake and large-scale development of this relatively complex and data-demanding model. The DSSAT crop modeling community is undertaking an effort to make its code open-source with the participation of more than 20 developers. The Global Trade and Analysis Project (GTAP) has provided extensive documentation of its model and data and allows user-modification of its standard model (Global Trade Analysis Project, 2014), and there is a large number of users of the model globally. The IMPACT model developed by the International Food Policy Research Center is publicly documented and available to other researchers (Rosegrant and IMPACT Development Team, 2012). The TOA-MD model for technology adoption and sustainability assessment of agricultural systems was developed based on experience which showed that potential users needed a user-friendly, transparent tool for impact assessment. The TOA-MD model is available to users with documentation and a self-guided learning course and there is a growing community of users (Antle et al., 2014b, Tradeoff Analysis Project, 2015).

To achieve the goal of demand-driven model development, it will be necessary to strengthen the linkages between the pre-competitive space of model development and the competitive space of knowledge product development. The current state of affairs appears to be that, on the one hand, the modeling community is strong on analytical capability but weak on linkage to user demand; while on the other hand, the developers of user-related farm-level products (e.g., providing data from mobile devices) are weak on analytics. Thus, there appears to be the opportunity for “gains from trade” by facilitating more interaction between the two communities. An important part of this interaction has to be to identify the key research that could enable better service delivery to knowledge-product users. Additionally, as emphasized in the NextGen Use Cases, there is a public good value to enhancing a broader community that can provide both data and analytics for public investment and policy decision-making. These ideas are further explored in the paper by Janssen et al. (this issue) and by Capalbo et al. (2017-in this issue).

### 2.3 New approaches to data acquisition, management and use

The explosion in the availability of many kinds of data and the capability to manage and use it create new opportunities for systems modeling at farm and regional or landscape scales. Fig. 2 presents an example of the possible types of private and public data that could be generated and used for both farm-level management Use Cases and for landscape-scale investment and policy analysis Use Cases. Some of these data would be generated and used at the farm-level, others would be generated and used for landscape-scale analysis to support investment decision-making and science-based policy-making. While farm-level decision making and landscape-scale analysis have different purposes, they both depend on two kinds of data: private data, including site- and farm-specific characteristics of the land and the farm operation, and the site- and farm-specific management decisions that are made; and public data, i.e. weather, climate, soils, and other physical data describing a specific location, as well as prices and other publicly available economic data.

Many farm-level data and decision tools from private and public sources are currently in use, and are evolving rapidly (Capalbo et al., 2017-in this issue). The left-hand side of Fig. 2 presents the generic structure of these tools, the data they use as inputs, and the outputs that are generated. The right hand side of Fig. 2 shows the general structure of the data and models needed to carry out landscape-scale research and policy analysis. A key feature of landscape-scale models is that they use public data for prices, weather forecast, and policy information, private site and farm-specific input use data, and outcome-based data that are useful for both farm-level management decisions and landscape-scale policy decisions.

There are three broad categories of landscape-scale data: publicly available bio-physical data, including down-scaled climate and soils data; publicly available economic data, including prices and policy information; and the confidential site- and farm-specific data obtained from producer- and industry-generated databases. Landscape management and policy analysis models require spatially and temporally explicit data that are statistically representative of the farms and landscapes in a geographic region in order to provide reliable information about economic and environmental impacts and tradeoffs. Such data are not typically available in most parts of the world. As a result, implementation of these models relies on the publically available information on farm management collected periodically through special-purpose surveys.

Currently available data are inadequate for various reasons. Many of these data are collected with samples that are not statistically representative of relevant regions or populations for landscape-scale analysis; many data are not spatially or temporally explicit, are only available (released) after substantial aggregation, thus limiting their usefulness, and are often available with long time lags between when the land management decisions are made, the data are collected, and when they become available for research or policy purposes. Longitudinal data that provide observations of the same farms over time are particularly important for policy research, but there are few such data available.

A key implication of the framework presented in Fig. 2 is the complementarity between knowledge product design, agricultural system models, and farm-level data collection. We return to this issue in Section 4.

### 2.4 A systems approach

The NextGen Use Cases show clearly the need for whole-farm system approaches. Agricultural systems are managed ecosystems (or agro-ecosystems) comprised of biological, physical and human components operating at various scales (e.g., cell, organism, field, farm). Farms are embedded within larger ecological and human systems operating at regional scales (e.g., watershed, population), as well as larger (continental, national, global) scales.

The need for a system-level understanding, however, should not be construed as meaning that there is not a need for component-level tools as well. Indeed, particularly in the more specialized, industrial systems, there will be a growing demand for tools to improve management of soil fertility, pests and diseases, and other elements of on-farm management. Nevertheless, until these components are integrated into a wider systems approach, it they will not be able to achieve goals of sustainable management. For example, nutrient and pesticide use cannot be managed effectively to account for potential off-farm impacts on water quality without a systems approach.

The systems approach has several important implications for second generation models. Within each system level, a set of interacting sub-systems is involved. This suggests the possibility of constructing models of large, complex systems by combining models of modular sub-systems. The level at which modularization may be possible remains an important question, and this in turn has implications for software engineering. For example, as discussed in Jones et al. (2017-in this issue B), many crops are now modeled individually and separate from livestock. Systems with multiple interacting crops (e.g., through rotations or inter-crops), livestock, and crop-livestock interactions, are needed for various Use Cases, showing the need for these interacting components to be incorporated in a modular “plug and play” system. Also, these biophysical production system components interact with economic-behavioral components and environmental components. These interactions among sub-systems show the need for standard ways to link inputs and outputs among sub-systems. As we noted above, several more complex systems models have been developed (Stoorvogel et al., 2004, Giller et al., 2011), but as yet each modeling system uses its own approach to model linking and model components from different developers cannot easily communicate with each other.

Another important issue raised by the systems approach is the appropriate level of complexity for Use Cases, an issue discussed further in Section 3.8. Research in environmental modeling indicates there are often diminishing returns to complexity. Similarly, experience with economic modeling has shown the value to “minimum data” or “parsimonious” approaches (Antle et al., 2014b). The need for both modularity and parsimony also relate to the need for generic approaches, particularly for complex agricultural systems models and economic models, so that model developers can move away from models that are application-specific.

Small-holder farm Use Cases illustrate the need for a systems approach at the farm level. In order to assess the well-being of the farm family in terms of income and nutrition, all relevant economic activities of the farm household need to be accounted for, including the income generated by the farming activities as well as other non-agricultural activities of the household members (e.g., off-farm work) and money transfers. Additionally, because the farm often involves multiple production activities, including crops and livestock, all of these activities and their key interactions need to be represented, as illustrated by the circular flow of nutrients from crops to livestock in the form of crops, crop residues and household waste fed to livestock, and then back to crops in the form of manure and composted materials.

The commercial-crop Use Cases 4 and 5 described in Antle et al. (this issue A) also illustrate the need for a systems approach. Crop rotations are important to the management of soil fertility and soil pests, and thus play a key role in achieving more sustainable management of input-intensive systems. It is also likely that to improve the sustainability of large-scale systems, it will be necessary to move towards more diversified systems that use crop rotations and integrate crops with livestock. The commercial-crop Use Cases also illustrate the need for assessments of landscape-scale impacts, including water quality (through soil and chemical runoff and chemical leaching), biodiversity (through impacts of fish and other wildlife), and greenhouse gas emissions (e.g., through soil management and fertilizer use). Similar types of assessment are needed to design and evaluate systems that meet the goals of “sustainable intensification” and “climate-smart agriculture.”

### 2.5 Credibility, uncertainty and model improvement

A clear message from actual and potential model users is that model credibility is a key issue limiting the use of models for decision making. This is an area where improved knowledge products could play an important role, by making it easier for potential users to understand the various uses of models and model outputs if they are going to interact directly with models and model output. Alternatively, as noted by Antle et al. (this issue A), many end-users of model outputs have no desire to interact directly with models, but rather want the information that models can provide. For example, many potential users think of them as predictive tools, and do not understand their use as exploratory tools in conjunction with future scenarios. Appropriate knowledge products can help users make appropriate interpretations and avoid mis-using models.

Transparent documentation and communications of model performance also has an important role to play in establishing model credibility, and could be facilitated by improved knowledge products linking data and models to users as in Fig. 1. There are potentially many different uses of models, from research to on-farm management to policy decision-making, and the criteria for a “useful” model differ among these. For some scientific purposes, a high level of precision may be needed, whereas for policy analysis, the timeliness of the information produced may be much more important than its precision or accuracy. Thus model evaluation calls for appropriate performance criteria, including overall model performance in providing outputs desired by end-users, as well as criteria for modular component improvement.

Several types of formal model evaluation techniques have been developed to assess complex systems model performance under current as well as future conditions. Evaluation under current conditions can be based on comparison with observed data through numerical, graphical, and qualitative methods, whereas assessment out of sample also involves the use of future scenarios which introduces additional uncertainties (Valdivia et al., 2015). A constructive recent example using an agent-based model that integrates bio-physical and economic models is provided by Troost and Berger (2014).

Another dimension of credibility is communication and interpretation of model uncertainty. Surveys of uncertainty methods are presented in Bennett et al. (2013) and Wallach et al. (2015). The more systematic use of methods such as global sensitivity and uncertainty analysis of future projections could provide a better understanding of the factors and underlying processes driving the numerical model output variance under particular scenarios that can be compared with conceptual models of the system (Saltelli et al., 2004). Statistical techniques also offer the opportunity to identify surprises in the future system behavior, as well as important feedbacks and non-linearities, and carry out a number of other evaluations of model behavior, model simplification, model inputs, and model uncertainty (Saltelli et al., 2005).

Model inter-comparison is another approach to model improvement that has been utilized in a number of scientific fields, including in the climate modeling field, through the establishment of the Coupled Model Inter-comparison Project (Taylor et al., 2012). By establishing protocols for the use of “reference scenarios” it is possible to inter-compare model results, identify important differences in model outputs, and through this process ultimately improve the models and their performance relative to the criteria described above. The use of model “ensembles” is also considered by some researchers as a way to characterize model uncertainty, although this interpretation is controversial. Model ensembles have been shown to perform better in some respects than individual models, suggesting the use of ensembles as a way to improve performance of agricultural systems models (Martre et al., 2014). A limitation of the ensemble approach is that it requires a relatively large number of alternative, independently developed models. In many cases, for example in economic modeling, there are not enough distinct models to make model inter-comparison or ensemble approaches useful. Also in economic models, different methods of quantifying model inputs and outputs makes inter-comparison problematic (von Lampe et al., 2014). Again, well-designed knowledge products could facilitate the use and interpretation of model ensembles to improve the appropriate use and credibility of agricultural systems models.

## 3 Potential advances in model components

The development of NextGen systems models will require numerous component improvements to address important Use Cases, as described in the companion paper by Jones et al. (2017-in this issue B). Here we discuss related cross-cutting issues as well as specific disciplinary component improvements that will be needed by NextGen agricultural systems models for the various major applications such as the five Use Cases as well as others.

### 3.1 Cross-cutting issues

A general cross-cutting theme addressed in Jones et al. (2017-in this issue B) and in Janssen et al. (this issue) is data generation and access. Lack of suitable data, or access to existing data, is a substantial impediment to model improvement and use. Here we focus on cross-cutting themes in model development.

#### 3.1.1 Representing and incorporating human behavior into agricultural systems models

Agricultural systems are managed by people for people. The objectives of the people using the information generated by models, and the behavior of decision makers whose behavior is represented in models, must influence model design. Most existing models have a limited capability to represent economic or other behavioral motivations of decision makers. This is a cross-cutting theme in modeling because the management decisions made by farmers are related to crop and livestock productivity, economic costs and returns, as well as environmental and social outcomes. There are several ways that behavior needs to be incorporated into NextGen models.

First, a better understanding of decision maker objectives is needed if we are to develop models that provide information on credibility for particular Use Cases, or that provide information to farm managers to improve decision making. For example, if production risk management is an important objective of decision makers, then they will need different kinds of information than if production risk is not a major issue. Note, however, that if the goal is to inform decision making, and not to predict behavior, then the behavior of the decision makers does not need to be modeled.

Second, for models that are designed to predict or project plausible outcomes or impacts of decisions made by farmers, the behavior of the decision makers must be modeled. This need adds a substantial complexity above and beyond the capability of modeling bio-physical production processes. Knowing what behavioral models are most useful for the Use Cases (e.g., profit maximization, risk management, achieving social status, other social or environmental objectives) is a key issue that needs to be addressed in NextGen model development.

Third, the social dimension of farmer decision making needs to be better understood and represented in models, including how social interactions and norms influence decision making. For example, technology adoption may be influenced by learning through social interactions. Modeling social interactions is an active area of economic research, but data demands are high and as yet empirical generalizations that could be used to structure models are not available. Other social scientists also study social interactions, but typically use qualitative methods that are difficult to translate into quantitative models.

#### 3.1.2 Representing heterogeneity

A key fact that has emerged from the increasing availability of field- and farm-level data is the high degree of biological, physical, economic and social heterogeneity of agricultural systems, in both space and time. The farms represented by the Use Cases demonstrate this point: among smallholder maize-based farms in Kenya, for example, coefficients of variation of key characteristics like farm size are on the order of 100% or more; for commercial crop farms in the United States, they are also large, ranging from 50 to 150%.

Heterogeneity has important implications for how we represent agricultural systems in models. Many modeling studies have utilized “representative farms” for typologies such as small and large farms in a geographic region (e.g., see van Wijk et al., 2014, Giller et al., 2011). These typologies can provide adequate approximations of heterogeneity for some purposes. However, accurate representation of bio-physical processes (e.g., crop growth, chemical leaching, erosion, chemical runoff) may require data showing the variation in site-specific conditions across the landscape in the form of a probability distribution. As an extreme example, Stoorvogel et al. (2004) showed the importance of variation within fields for analysis of erosion and chemical leaching in steeply sloped hillside agriculture. This is also true for analysis of economic distributional issues such as changes in poverty rates that requires a model capable of estimating the income distribution.

#### 3.1.3 Representing dynamics

Agricultural systems are inherently dynamic: crop growth occurs over time within the growing season; crop productivity may depend on soil moisture and nutrient carryover, crop rotations and other dynamics of the system. Consequently, most process-based bio-physical simulation models of agricultural systems are dynamic, with associated needs for data both over space and time to represent both spatial heterogeneity and temporal properties (Jones et al., 2017-in this issue B).

However, the problem of representing human behavior in a dynamic setting adds greatly to the complexity of systems models (Antle and Stoorvogel, 2006). Economic behavior depends on expectations of future outcomes, and decisions are made sequentially, with information being acquired as decisions are made and realizations are observed. Some management decisions like fertilization rates are based on intra-seasonal processes (getting the highest profit that season); other longer-term decisions span multiple growing seasons (multi-season crop rotations; machinery investments; livestock purchases and sales, and perennial crop planting and management decisions). Similarly, it is challenging to represent both dynamics and heterogeneity in economic models, and most dynamic models are highly simplified or stylized. The challenge is even greater when multiple dynamic model components are linked, due to differences in spatial and temporal units and overall model complexity.

An area where there is a particular need for advances in the dynamic analysis capability is for assessment of the impacts of weather variability and extremes on agricultural systems, and the search for more resilient systems better adapted to such conditions. Current impact assessment methods that use averaged weather data, or economic analyses that do not incorporate dynamic effects of extreme events on a farm household's wealth and assets such as livestock, are not able to effectively evaluate these questions.

Although it is beyond the scope of this paper to elaborate on the challenges of modeling system dynamics, it is clear that progress in modeling system dynamics is essential but challenging. How to achieve this progress in a tractable and useful way should be a priority for basic research on next generation models, but motivated by the kinds of uses that stakeholders see for models and related knowledge products.

#### 3.1.4 Pathway and scenario design

It is not possible to model everything that influences an agricultural system. Consequently, models are based on a logical structure in which some factors (“drivers” or “exogenous” variables) take on values specified by the modeler or the model user. How these drivers are set or modified to represent the conditions under which the analysis is being carried out is a key aspect of modeling that has been under-studied. The issue is now receiving more attention in climate research, but needs to receive more attention from the wider modeling development community. In particular, if models are to be linked to end-users through knowledge products, the user needs to understand the context in which the analysis or “simulation experiment” is being conducted and the types of effects that are being quantified (Antle and Stöckle 2016).

One solution to this challenge is through a participatory process of pathway and scenario development, building on the participatory modeling methods that have been developed, e.g., see Giller et al. (2011). For climate impact assessment, AgMIP has been developing systematic approaches to development of “pathways” (plausible future conditions) and “scenarios” (specific parametric representations of a system consistent with a pathway), using the concept of Representative Agricultural Pathways (Valdivia et al., 2015). Stakeholders can provide inputs into the future pathways and scenarios, and this participation also facilitates their ability to use model outputs. An example of a knowledge product could be “apps” that help users of climate impact assessments better understand climate assessment scenarios and better interpret model results. AgMIP is developing an “impacts explorer” as a type of knowledge product for this purpose. New approaches are now being explored for scenario development that exploit new data and advanced computer science methods such as machine learning.

### 3.2 Crop systems

Development of next-generation crop models can be divided into several categories: significant improvements in simulation of important crop processes and responses to stress; extension from simplified crop models to cropping systems models that address complexity in space, time and the number of processes considered; and scaling up from field-based models to landscape, national, continental, and global scales.

#### 3.2.1 Needing improvements

Several crop processes require major advances in understanding and simulation capability in order to narrow uncertainties around how crops will respond to changing atmospheric conditions, both changes in mean variables and changes in extremes. Experimentalists and modelers need to work together from the outset to ensure that the right research questions are posed as experiments are planned, critical field data are gathered at appropriate times, and process-based understanding is captured so as to transfer new insights from the field to the crop models directly and expeditiously.

##### 3.2.1.1 Extreme events

Extreme weather and climate events are responsible for significant economic and social costs in agricultural regions around the world and are expected to increase in duration and intensity under most climate change scenarios (IPCC, 2014). Crop models need to accurately represent the relevant impacts of weather and climate extremes. This includes more precise understanding of what thresholds qualify a weather or climate event as “extreme” for different crops in different regions and what simulation processes need to be improved to describe crop responses and their variability to such extreme events. Sequential periods of yield-reducing weather conditions can be especially damaging, such as two or more consecutive dry years as often experienced in sub-Saharan Africa (e.g. Tittonell, 2014). Other extreme events could be extended periods of record-high temperatures or flooding during a growing season.

##### 3.2.1.2 Genetics

Developing predictive capacity that scales from genotype to phenotype is challenging due to biological complexities associated with genetic controls, environmental and management effects, and interactions among plant growth and development processes. Crop model improvements are needed to link complex traits at gene network, organ, and whole plant levels (Parent and Tardieu, 2014). Phenotypes are linked to changes in genomic regions via associations with model coefficients (Hammer et al., 2006). Hwang et al. (this issue) discuss how genetic information can be incorporated into next generation crop models.

##### 3.2.1.3 Carbon, temperature, water, and nutrients

Crops are already experiencing higher levels of carbon dioxide (CO_2_) and temperature in agricultural regions around the world. Understanding of how accelerated rates of CO_2_ and temperature rise will interact to affect crop growth and productivity is growing (Long et al., 2006), but this improved understanding needs to be incorporated into crop models (Leakey et al., 2009). Water relations of soils and crops are also important, and optimizing carbon and nutrient cycling, as well as multiple nutrient interactions – beyond the current focus exclusively on nitrogen x water interactions – plays a crucial role in sustainable intensification. The simulation of all of these processes and their interactions and management, especially (i) under conditions of stress and (ii) considering soil biology-mediated processes, needs to be radically improved.

##### 3.2.1.4 Pests, diseases, weeds, and their management

Insect pests, diseases and weeds are important yield-reducing factors in terms of food production and economic impact, but are not addressed adequately or at all in most models. This important challenge is addressed in Section 3.5 below and in Donatelli et al. (2017-this issue).

##### 3.2.1.5 Ozone

There is evidence indicating that ozone damage to crop productivity is substantial, but is rarely considered in crop modeling studies (Leisner and Ainsworth, 2012). Information about ozone impacts on crop yields is available, but damage processes and functions need to be developed. Model improvements in regard to ozone effects on crops include inclusion of ozone response functions and comparison of response functions with process-based approaches such as leaf conduction, aerodynamic boundary-layer resistance, and whole canopy conductance parameterizations. In order to learn much more about the different responses of different crop species and varieties, ozone data collection should be incorporated into the AgMIP protocols for sentinel site experimental design.

##### 3.2.1.6 Nutrition

Crop modelers, breeders, physiologists, and human health and nutrition researchers need to broaden the scope of modeling to include nutritional quality of food as well as behavioral aspects of food utilization so as to enable more fully developed projections of future risk of hunger due to climate change and development pathways. For crop modelers, this requires moving from a yield-only perspective to one that includes processes that affect nutritional quality, such as carbon dioxide concentration, drought, and insect pressure (Rosenzweig and Hillel, 2008). Simulations of non-staple crops, for many of which crop models have not been developed, are needed to understand nutritional effects (Müller et al., 2014). For example, as people move out of hunger, one of the primary correlates of health is fruit and vegetable consumption and better models of how these crops may be affected by climate change and other processes will become increasingly important. At cropping system level, there is mounting evidence on the strong positive correlation that exists between crop diversity, nutritional functional diversity and balanced diets in developing regions (e.g. Khoury et al., 2014, Remans et al., 2011).

#### 3.2.2 From crops to farming systems

The field of crop modeling was built on a single crop-by-crop approach. A new paradigm is now emerging that moves beyond ‘crops’ to ‘farming systems.’ These new farming system simulation tools incorporate the complexity that comes with many interacting biophysical and socioeconomic components (van Wijk et al., 2009, Giller et al., 2011). Such farm system models, however, have tended to place emphasis on farm-scale interactions between system components while reducing the detail with which cropping systems are simulated, to reduce uncertainty and numerical dispersion, by means of so-called ‘summary models’ (e.g. Tittonell et al., 2010) or more parsimonious or ‘minimum-data’ approaches (Antle et al., 2014b). Such tradeoffs between model complexity and modeling capabilities can be now better overcome through the increasing computational power and data available for NextGen models. From the crop modeling perspective, progress on the number and types of simulated crops (including vegetables and fruits), uncertainty propagation related to model parameters and structure, *ex ante* testing of adaptations, and scaling are needed (Ewert et al., 2015a).

##### 3.2.2.1 Intercrops and complex rotations

Many models now incorporate the ability to simulate multi-year crop rotations (e.g., Stöckle et al., 2003, Kollas et al., 2015). Some models allow for more than one crop in the same field. For example, Corre-Hellou et al. (2009) developed a model for pea–barley intercropping based on STICS that allows an inversion of dominance in height between species during the crop cycle and a trophic link between crop growth rate and the potential for N_2_ fixation. Next steps in regard to rotations and intercrops are to advance technology so that modelers can rapidly incorporate multiple crops within fields, and multiple crops over time as the usual practice. Then the response of these more complex cropping systems can be tested under different sustainable intensification management strategies utilizing the updated simulation environments. Similarly, inversion studies can be performed to determine optimal cropping systems and management strategies for particular desired outcomes.

##### 3.2.2.2 Linkages to livestock production

Most smallholder farming in the world involves integrated crop-livestock systems that cannot be represented by crop modeling alone. Thus, next-generation farming system models include key linkages to livestock. Progress towards these linkages has been made in the NUANCES-FARMSIM (Nutrient Use in Animal and Cropping systems: Efficiencies and Scales FARM SIMulator) that allocates limited resources across the farm and simulates the way organic matter is recycled or redistributed within the farm in both crops and livestock (Tittonell et al., 2009); these decisions determine the long-term production capacity of the system (van Wijk et al., 2009, Giller et al., 2011). Valdivia et al., 2012, Valdivia et al., 2016 developed and applied a bio-physical and econometric simulation model that includes dynamic interactions between crops and livestock through nutrient cycling.

As discussed below, livestock linkages that need to be fully incorporated include growth and productivity models for perennial grasslands and rangelands as well as the usual annual crops used as fodder. Modeling of grassland and rangeland systems requires also considering the grazing/browsing behavior of herbivores and their interaction with grass/range species, which typically leads to spatial heterogeneity in productivity and other ecosystem services. Information from local experiments (such as collated by the Global Research Alliance - Sandor et al., 2016) will be required to develop and test the grassland and rangeland models in a wide range of environments. These models will then be capable of deployment with livestock models, regional farm data, and inputs related to management and climate. On the management side, the effects of animal labor need to be included as well.

#### 3.2.3 Scaling up from field to landscape

New farming system simulation tools are incorporating the complexity that comes with many interacting biophysical and socioeconomic components, especially in smallholder farming systems in developing countries. A key issue is how to represent heterogeneity in these complex systems; some modeling frameworks use “typologies” (van Wijk et al., 2009, Giller et al., 2011), while others take a distributional, point-based or gridded approach that may represent the range of conditions more fully (Antle et al., 2014b), if data needed to characterize those variations are available. The question of appropriate model detail is clearly important (Giller et al., 2011).

For the cropping portions of the more complex farming systems models, future research should focus on improving crop models for larger-scale applications. To date, large-area crop models have not been developed to capture the relationships important at an aggregated regional scale and long time-horizons (Ewert et al., 2015b); the AgMIP Coordinated Global and Regional Assessment (GCRA) is undertaking this task (Rosenzweig et al., 2016). Other areas for research aimed at better understanding of scaling-up of crop models for large-area assessments include: inclusion of spatial variability in soils (Laloy and Bielders, 2009) and in management, particularly for N fertilization, sowing dates, and crop varieties. A particular challenge is to understand the impact of methods to scale-up crop rotations (Teixeira et al., 2015).

Cropping system models need to be able to simulate easily a diverse set of farms rather than just one or several representative farms. There are several approaches for scaling up, including use of gridded models and development of simpler quasi-empirical models for landscape-scale analysis (Lobell and Burke, 2010, Ewert et al., 2011). Large-scale computation can allow for much more extensive use of gridded models than in the past (Elliott et al., 2014, Jones et al., 2017b). Soils and climate input datasets become important as simulation goes from field to landscape scale. There are several types of dynamic process gridded crop models: those developed from the site-based models such as DSSAT and APSIM; ecosystem-based models; and dynamic land-surface models. An example of a more statistical model is the agroecological zone (AEZ) approach developed by IIASA and the FAO (Fischer et al., 2000.

Landscape processes such as biotic interactions between pests and their natural enemies in space and time are currently poorly captured in cropping system models. Attempts to model landscape level population dynamics for both pests and bio-controllers tend to simplify cropping systems and their dynamics, and consider them as different ‘land uses’. Yet the diversity and phonological stages of crops in the landscape, plus the management practices implemented in each field, have an important influence on the population dynamics of crop pests and diseases. Much progress is needed in linking these two scales in our modeling of agricultural landscapes, aiming to contribute to their sustainable intensification.

#### 3.2.4 Crop model interoperability and improvement

A key question for the next generation of cropping system models is the degree of interoperability. Historically, scientists (as individuals or groups) tended to have exposure to, and in-depth knowledge of, a single crop model (Thorburn et al., 2015).

Crop models allow useful extrapolation and prediction for prescriptive management, but most current crop models lack the ability to handle spatially connected processes (i.e., water flow, weeds, and pest dynamics) within a field or landscape. Use of the models with real-time, remotely sensed data is not currently available to most farmers or farm advisors (Basso et al., 2001, Janssen et al., 2016).

AgMIP aims to increase efficiency of model improvement and application by sharing information between different models and encouraging the use of multiple models in impact assessment (Rosenzweig et al., 2013). Ideally, parameters from one crop model can be uploaded into databases and then downloaded, reformatted for use in another model. However, AgMIP has found that this sharing of parameter values between models is not necessarily straightforward.

AgMIP is bringing different modeling groups together to compare and improve their models. The aims are to develop a better understanding of different crop models across the agricultural modeling community; improve both individual crop models and the entire group of models for a particular crop; and improve the efficiency and effectiveness of multi-model applications in agriculture.

### 3.3 Livestock production

In addition to the linkages with crop models discussed above, including the need for modularization and inter-operability, there are a number of areas in which advances in livestock modeling could improve the information needed to support a variety of Use Cases, for both farm-level and landscape-scale decisions. More comprehensive livestock models are needed, covering a wide diversity of ruminant and other species, adequately pre-parameterized for most common situations and with default values for users to parameterize models to their conditions. Summary or meta-models from comprehensive, dynamic models could be developed as on-farm decision aids. These tools could include summary models for intake, production and greenhouse gas emissions calculations. Some of these summary models could be developed as mobile phone technologies.

Other improvements could include development of extensive, standardized feed libraries linked to a GEO-WIKI for improving mapping of feeds globally. These libraries could also be used for deriving functions of feed quality for different agroecological conditions. One way this could be accomplished would be to expand existing household data collection protocols to include suitable data for livestock.

As a step towards addressing heterogeneity, more detailed crop and livestock production systems typologies would be useful. These typologies could be derived from existing farm household, agro-ecology, farm, rangeland, population, markets and other spatial data. NextGen production systems mapping needs to include intensification, gender dimensions of family labor and control over assets and income, and operation size indicators.

Better spatial data are needed, including spatially explicit standardized feeds and productivity data. Ideally these data would be linked to crowdsourcing and large data rescue initiatives. Improved spatially explicit farm and regional data on production costs for different livestock technologies are also needed. This information is seldom available and is crucial for both regional and global analyses. These data would enable bio-spatial analysis of livestock yield gaps to guide investments and to identify opportunities to use livestock as a vehicle for agricultural development, poverty reduction, and environmental protection.

Livestock components of future scenarios are needed for climate impact assessment and other forward-looking analysis. Improved and consistent story-lines are required for the livestock sector in all scenarios. These story-lines can be produced as part of global and regional “representative agricultural pathways” being developed by AgMIP and other research teams. Currently, such story-lines exist only for the global “shared socio-economic pathways” used in climate impact assessments; see Havlik et al. (2014); Herrero et al. (2014).

### 3.4 Pastures and rangelands

Pastures and rangelands are integral to all livestock production systems and are often closely integrated with crop production systems (e.g., pasture in rotation). The biophysical components of these systems and driving data required to model them are largely similar to those of crop production systems, but several features of these components of agricultural systems need to be addressed in next generation models.

Management data tend to be sparsely available and representing continuity of plant populations is challenging. Advancing our ability to understand how grasslands are managed – to understand, for example, what species are planted, what inputs (irrigation, fertilization, etc.) are provided, what grazing management (timing, intensity) is applied – is centrally important for improving our ability to model pasture and rangeland systems. Planted pastures and native grazing lands both contain a variety of species, some of which are more palatable, nutritious, grazing-resistant, or fire-resilient than others.

A more open, data-rich environment could facilitate evaluation of a variety of approaches for representing long-term dynamics, which could address several important grassland management and assessment issues. Managing grass swards (and desirable forb and species) to maintain desirable plants is a primary goal of grassland management, but one for which modeling tools have offered limited assistance. Models that represent vegetation dynamics are also desirable for understanding longer-term changes in species that can impact productive capacity, sensitivity to degradation, and carbon dynamics (particularly woody encroachment). Year-to-year variability is a key component for understanding potential utility and risk of relying on grassland forage resources. Next generation models that enhance our ability to forecast this risk would mark a substantial and meaningful advance. There is a need for better links between the agricultural modeling communities and ecological researchers studying long-term vegetation dynamics.

The primary use of forage resources is for grazing animals, yet most grassland models are only loosely coupled with grazers (livestock or wildlife). Better integration through grazing effects on grasslands, grazer distributions across landscapes, forage demand/consumption, livestock/wildlife movement, etc., would enhance the ability of models to contribute to important emerging issues. For example, holistic grazing management, in which several aspects of management vary in response to a variety of different cues from the land and expectations about future conditions, can be impossible to evaluate with current modeling frameworks. A system that integrated user demand into the model development process could lead to implementation of new data-management feedback loops within models. Such interactions between users and producers of information could direct data collection (e.g., by drone or remote sensing) to facilitate model use. Models that better represent grazer-grassland interaction are also crucial for understanding how efficiently livestock use forage resources, what is necessary to sustain wildlife populations, and how much grassland output might be available for other uses (e.g., biofuels).

### 3.5 Pests and diseases for crops and livestock

As noted above, a major limitation of existing models is how they represent pests and diseases. A number of limitations and needs for pest and disease models and their use in crop and economic models were presented in a recent review by van Bruggen et al. (2015) and are elaborated in Donatelli et al. (2017-this issue). Here, we note some of the important areas that must be improved in NextGen models.

#### 3.5.1 Improved statistical modeling of within-season pest and disease threats using automated data collection and cloud computing

It is now possible to collect weather data continuously from ground-based sensors and to merge these data with medium-term weather forecasts and remote sensing data on crop growth and pest and disease damage. (Both growth and damage can be detected by satellite or drone by monitoring the crop's spectral properties.) Then, using sophisticated statistical modeling done centrally, real-time advice can be distributed to farmers through the web or through mobile phones enabling them to take precautionary actions.

#### 3.5.2 Understanding the consequences of climate change for weed, pest and disease threats

The Intergovernmental Panel on Climate Change has reviewed the existing evidence for how climate change may affect weeds, pests, and diseases (Porter et al., 2014). One issue with this evidence base is that there is a clear publication bias towards reports of increased threats – people often do not bother to write up no-effect results. There is a general recognition that we need good models to help tease out different effects that changing weather will have simultaneously on both crops and the organisms that compete with or attack them. There has already been some work applying crop physiology-type models to weeds, and developing more mechanistic models of the effect of temperature on insect pests. There is an opportunity and need for more integrated models that include interactions between organisms, for example between weeds and crops, and between pests and the predators and parasites that attack them. A variety of different approaches are possible, and there is a need for an AGMIP-type approach to help the community decide how best to move forward.

#### 3.5.3 Livestock disease

Highly contagious diseases of livestock present a major threat to agriculture, both in the developed and developing worlds. Diseases may be chronic in livestock populations, emerge from wildlife reservoirs, or possibly be introduced deliberately by man as an act of bioterrorism. Models are required to help understand how a disease will spread, and to help policymakers design optimal interventions. These models must encompass not only the epidemiology of the disease but also how it is affected by agricultural practices and in particular the movement of livestock by farmers. There have been significant recent advances in this area, often building on work on human diseases. For example, it is now possible to take livestock movement data and use it to parameterize an epidemiological model (Kobayashi et al., 2007, Brooks-Pollock et al., 2014). There are the beginnings of a model comparison movement in human epidemiology; livestock disease epidemiology would also benefit from this approach.

#### 3.5.4 Novel genetic control methods

There is intense current research activity into novel genetic methods of insect control. Most of this work is currently directed at the insect vectors of human diseases such as malaria, though the same methodology can be applied to insect pests of crops and of course the vectors of livestock diseases. The greatest advantage of these approaches is that they involve self-sustaining interventions that spread naturally through a pest population, although because they are nearly all classified as genetically modified, the regulatory issues surrounding them are complex. Cutting-edge modeling work in this field involves joint population and genetic dynamic models, many of which are explicitly spatial. This topic is likely to be one of the most important and exciting areas of modeling as applied to agriculture over the next few decades.

### 3.6 Precision management, data and information technology

Integrated agricultural technologies, defined as the integration of improved genetics, agronomic input, information technology, sensors, and intelligent machinery, will play a pivotal role in agriculture in the years to come. These innovations will be driven by economic forces, by the need to produce more food with limited land and water for the increasing population, and at the same time by the push to save resources to reduce the environmental impact associated with food production. While these changes are occurring now in the commercial-scale industrialized agricultures of the world, many of these technologies have the capability to be adapted to conditions in other parts of the world. The cell phone now allows farmers in rural areas almost everywhere in the world to have low-cost information about prices, for example. Similarly, it is likely that unmanned aerial vehicles will rapidly be adapted to conditions around the world and used to carry out activities such as monitoring crop growth and pest occurrence, and improve management decisions. In large-scale, capital-intensive agricultural systems, these technologies are rapidly leading to the automation of many production activities, particularly machinery operation and decisions about input application rates.

The automation of agriculture began in the mid-nineties, resulting in large amounts of data available to farmers and agribusiness companies. Farm machinery are now often equipped with high precision global positioning system controllers, which allow all activity on the farm to be recorded, geo-referenced, and stored on remote computers: “in the cloud.” All modern tractors collect data on a continuous basis and are equipped with wireless connectivity for data transmission. Harvesters record the yield at a particular location, planters can vary the plant spacing or type of seed by location, and sprayers can adjust quantity and type of fertilizer, fungicide or pesticide by location; all to a granularity of just a few square meters. Yield monitoring can now be linked to unmanned aerial vehicle (UAV) imagery to produce a prescription map for the farmer to implement. These private data could also provide tremendous benefit to the researcher community, should access be increased.

Producers in some regions of the world now have historical crop yield data for their fields at very high resolution. Combined with advanced satellite-based imagery, high-resolution spectral and thermal data obtained from UAVs, and weather forecasts, growers have most of the critical inputs required to convert this “big data” into an actionable management plan with equipment that can vary fertilizer and other inputs spatially within a field. Despite these rapid advances in the sophistication and automation of farm equipment, a vital piece of the equation is still lacking: the analysis of the vast amount of newly available data in order to provide the farmer with a map of what action to take where and when. Most variable rate application is currently managed by farmers, using rule-of-thumb and empirical approaches, and not by using a systems approach that accounts for the interaction of soil, crop, management, and weather. Thus much of the power of automation remains unexploited.

In order to realize the full potential of more sophisticated equipment, new modeling systems for precision agriculture are needed. These systems could be based on comprehensive predictive crop yield models that combine publicly available data, such as soil type, weather, and prices, along with location-specific data from farmers' yield maps of their fields, to provide a prescriptive crop management plan at high spatial resolution, as in Fig. 2. This type of system could deliver automated crop simulations, crop management strategy recommendations, process-based variable rate prescriptions, risk assessments, continual in-season simulations, integration of in-season crop scouting UAVs flight information, pest management prescriptions and accurate harvest recommendations via simple-to-use apps, websites, or smart phones.

**Fig. 3:**
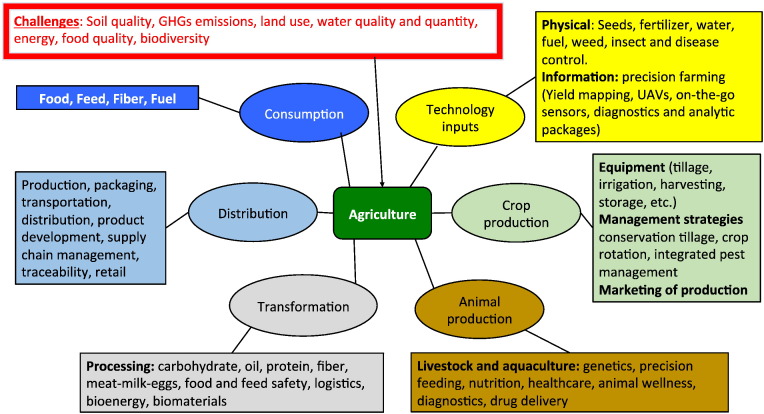
Components of the agricultural sector value chain.

In addition to the farm-to-landscape scale analysis represented in Fig. 2, there will be a growing demand for agricultural systems models to simulate and integrate the different components of the agricultural value chain, to meet both policy requirements and corporate sustainability goals (Fig. 3). Genetics, agronomic management (production input), weather, soil, information technology and machinery will need to be linked in a system approach to address these informational needs. This is a new frontier for agricultural system modeling that would extend to the broader food system and raise additional data and analytical challenges.

### 3.7 Economic and social dimensions

We have noted earlier that the economic-behavioral aspect of agricultural systems and their management is an area needing more attention, as suggested by a number of Use Cases at the farm and regional or landscape scales.

#### 3.7.1 Farm-level decision support

As illustrated in Fig. 2 and discussed in the previous section, various management and production data are becoming available through mobile technologies (e.g., tracking soil conditions, seeding and fertilizer application rates, pesticide applications, crop growth, yield). An example of this analytical capability is the AgBizLogic™ software developed by several university extension programs, which allows managers to calculate short-term profitability and rates of return on long-term investments (Capalbo et al., 2017-in this issue). Similar proprietary software tools are being developed and used. These analytical tools could be linked with modules that track or predict environmental outcomes such as soil erosion and net greenhouse gas emissions (e.g., AgBalance by BASF). Low-bandwidth versions of these tools need to be developed for use in areas where mobile phone technology is a limiting factor. Analytical tools need to be adapted to fit small-holder systems as indicated by the NextGen Use Cases.

The flood of data on physical land-use, water availability and use, and yields coming from mobile devices and remote sensing systems suggest that both the biophysical and behavioral aspects of farm production at specific locations can be estimated by sequential learning processes. The use of advances in computational methods such as machine learning and remote sensing data is illustrated by analysis of the impact of the 2009 and 2014 droughts on California agriculture, which demonstrated the advantages of better data (Howitt et al., 2014).

#### 3.7.2 Model improvements for regional investment and policy analysis

To facilitate the use of models for various locations and systems, and to link to crop and livestock system simulation models, economic models need to be incorporated into modules with standardized inputs and outputs. Various types of economic models are available in the literature, including farm-level optimization models, regional positive quadratic programming models, econometric land-use models, and regional impact assessment models (van Wijk et al., 2014). User needs should dictate which types of models should be used depending on informational needs.

Methods and protocols are required to link regional economic models (price-taking land use and impact assessment models) with market equilibrium models (e.g., regional partial or general equilibrium models). Some progress has been made on this front (Valdivia et al., 2012, Van Ruijven et al., 2015) but much more development is needed to address various aggregation and dis-aggregation issues (Antle et al., 2014b).

Generalization of behavioral assumptions and investigation of their effects on investment and policy analysis is also needed. Most economic models make simple profit maximization assumptions. There is a rich literature on risk modeling which could be incorporated. Recent advances in the expectations formation literature and the behavioral economics literature could be investigated for use in agricultural systems models.

#### 3.7.3 Social dimensions

As noted in Section 2, a demand-driven approach is needed that begins with user-identified outcomes and indicators. Various outcomes are of interest in the context of sustainability, and users often are seeking to understand tradeoffs and synergies among economic, environmental and social dimensions. Here we identify some key outcomes that need to be incorporated into modeling approaches to address various Use Cases.

##### 3.7.3.1 Income distribution and poverty

Most economic models provide an estimate of some components of farm household income, but a complete characterization of income sources is needed to evaluate income distribution and poverty in farm household populations. Population-level outcomes are needed, not only means or averages, as noted in the discussion of heterogeneity in Section 3.1.2.

##### 3.7.3.2 Food and nutritional security

Existing models represent food production, but no existing model characterizes all factors that affect food security (availability, access, stability, utilization) at the household or regional levels. A major limitation is data on food consumption at the household and personal levels over time. New methods of collecting these data using mobile devices are being developed. Additionally, it is necessary to express these data in other nutrient currencies beyond kilocalories, in order to explore nutritional diversity issues, as well as sustainable diets (Müller et al., 2014).

##### 3.7.3.3 Health

Earlier work on health impacts of pesticide use on farm workers and other occupational risks could be used to construct health impact modules (Antle and Pingali, 1994). As elsewhere, big data (e.g., in this case, data from medical records or insurance claims) can be used to improve understanding of impacts (Rzhetsky et al., 2014). Specialized health outcomes models can be linked to landscape-scale and global-scale models (Springman et al., 2016).

##### 3.7.3.4 Age, Gender and Health Status

Research on various aspects of gender impacts and outcomes has advanced, primarily in terms of relevant measures. With better data, analysis of gender impacts associated with new technologies could be incorporated into existing farm household models and impact assessment models. A similar situation exists for analysis of impacts by age and health status.

##### 3.7.3.5 Vulnerability and equity

The application of different farm improvement methods has explicit winners but also unintended ‘casualties’ and perverse incentives. From a development standpoint, it is essential to understand these dynamics to ensure that appropriate policies are developed to maintain equal opportunities for all sectors of society. For example, in many cases, rich farmers are the ones who adopt technologies early. This factor could potentially disrupt power relationships in markets, thus affecting poorer farmers. In this case it is essential to design alternative options and safety nets for poorer farmers to prevent widening the gap and making them more vulnerable. New models should improve our understanding of these processes, as we move from single farm models to multi-farm and regional models. Methods utilizing population-based data are providing improved capability to represent distributional impacts and vulnerability (Antle et al., 2014b, Antle et al., 2015).

### 3.8 Environment and system complexity

**Fig. 4:**
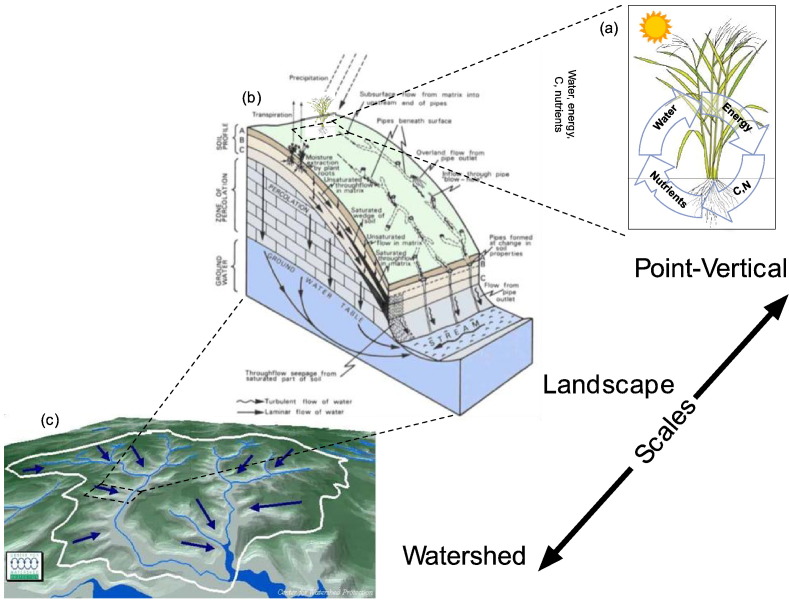
Lateral connections across scales and environmental components.

Current agricultural system models typically operate at the point/field scales (Fig. 4a) with an emphasis on vertical fluxes of energy, water, C, N and nutrients between the atmosphere, plant and soil root zone continuum. A holistic upscaling from the point source to the landscape scale (Fig. 4b) requires incorporation of several interacting, complex components, adding substantial complexity above and beyond the agricultural system itself. Thus, a major consideration in environmental modeling is how to best capture essential interactions while maintaining models that are feasible to implement with available data and computational resources.

Fig. 4 illustrates the various components linking point to landscape scales. A first element for the linkage from point to landscape is estimation of surface and subsurface fluxes and ecological transitions along the lateral scale. Coupling with landscape microclimate models provides the vertical inputs used by the agricultural systems models, as well as gradients (precipitation, temperature, wind, vapor pressure deficit) along the landscape. Coupling with hydrological models provides water flow paths like surface runoff, vertical and lateral groundwater flow, and interactions between vadose and groundwater zones and with adjacent surface water bodies (channels, rivers, lakes and coastal waters). Water quality models provides sediment and solute transport along the landscape controlled by water flows (Fig. 4b), and other effects like wind erosion. Integration and upscaling of landscapes into the watershed scale (Fig. 4c) requires 3-dimensional coupling of the surface and subsurface water, energy and mass transfers. At this scale, the groundwater aquifer system typically transcends the boundaries of the watershed and necessitates analysis at the regional scale to evaluate not only the impacts of the cropping and animal production systems on water quantity and quality, but also feedbacks from the hydrological system in the agricultural system (shallow water table effects, drought or low water availability for irrigation). Further, mesoscale rainfall and evapotranspiration distribution models control the local surface and subsurface flow intensities, pollution and abatement. At this scale, human effects through land-use changes as well as ecological (vegetation, wildlife) dynamics and transitions on natural or protected lands (riparian zones, conservation areas, water resource management infrastructure etc.) are also an important and critical component to evaluate the overall sustainability of the agricultural system.

Current crop modeling upscaling approaches based on land use maps can be considered an efficient first-order approximation of the environmental linkages. For example, in the USA the US Geological Survey (USGS) hierarchical system of Hydrologic Units Codes (HUC) (Seaber et al., 1987) is commonly used as the reference spatial mapping system to link spatially-explicit hydrological and crop yield simulations. Srinivasan et al. (2010) applied the Soil and Water Assessment Tool (SWAT) model (Arnold et al., 1998) to 8-digit *subbasin* HUCs (each with average surface area of ~ 1813 km^2^) in the Upper Mississippi River Basin and compared yields of the main crops (corn and soybeans) with observed county-level USDA National Agricultural Statistical Survey (NASS, www.nass.usda.gov/Data_and_Statistics/Quick_Stats/index.asp) data obtained for 1991–2001. SWAT uses spatially distributed watershed inputs to simulate hydrology, sediment and contaminant transport and cycling (pesticides, bacteria, and nutrients) in soils and streams, and crop/vegetative uptake, growth and yields. Because many counties in the NASS database have missing data it was necessary to aggregate the crop yield data and simulation results to 4-digit *subregion* HUCs (each with average surface area of ~ 43,511 km^2^). In general SWAT predicted crop yields satisfactorily over the long-term average for most 4-digit HUC (yield error less than 15%), although errors greater than 20% were found for 14% of the HUCs studied. Further information on crop management (e.g., fertilizer, tillage, and harvest) may improve SWAT's perform conclude that these errors stem likely from those predicting AET and soil moisture storage at these large aggregated scale, and “one could extend the validity and confidence in the model prediction of AET and soil moisture using a well-compared model on crop yield” (Srinivasan et al., 2010). Thus, next generation models should consider the lateral connections through the landscape and regional scales to evaluate the sustainability of the integrated system, including effects on water and soil resources quality and quantity and ecological value.

Although model complexity has increased in recent years and is a natural outcome of the proposed next generation integrated modeling, there has been little work to rigorously characterize the threshold of relevance in integrated and complex models. Formally assessing the relevance of the model in the face of increasing complexity would be valuable because there is growing unease among developers and users of complex models about the cumulative effects of various sources of uncertainty on model outputs (McDonald and Harbaugh, 1983, Manson, 2007, Cressie et al., 2009, Morris, 1991). New approaches have been proposed recently to evaluate the uncertainty-complexity-relevance modeling trilemma (Muller et al., 2011), or to identify which parts of a model are redundant in particular simulations (Crout et al., 2009). Innovative approaches to simplify model outcomes to make them relevant in decision-making will be central to the next generation modeling efforts. New methods for evaluating uncertainty also can be used to devise model simplification strategies. For example, the identification of processes that do not influence particular scenarios, and the use of meta models, could allow simplification without affecting results (Ratto et al., 2007, Villa-Vialaneix et al., 2011, Ruane et al., 2014).

## 4 Conclusions

We envision new models and knowledge products that could accelerate the innovation process that is needed to achieve the goal of achieving sustainable local, regional and global food security. Building on the analysis of a set of Use Cases and interactions with stakeholders, we propose a user-driven approach to agricultural system model development that would link a collaborative “pre-competitive space” for model development and improvement to a “competitive space” for knowledge product development. In addition, we identify desirable features for models, and describe some of the potential advances that we envisage for model components and their integration.

The concluding article for this Special Issue explores an implementation strategy that could link a pre-competitive space for model development to a competitive space for knowledge product development (Antle et al., this issue B). This strategy involves critical advances in data, and model developments at multiple scales. A key element is engagement of stakeholders through research relevant to major Use Cases, including small-scale systems that dominate developing country agriculture, industrialized agricultural systems, and analysis for major policy challenges such as climate change mitigation and adaptation. Specific model improvements, such as those discussed in this paper, would be based on further testing and evaluation of existing models, the development and testing of modular model components and integration, and linkages of model integration platforms to new data management and visualization tools.
